# The Hospital Sunday Fund

**Published:** 1911-04-01

**Authors:** 


### THE HOSPITAL SUNDAY FUND.

### THE DISCUSSION ON SUNDAY CINEMATOGRAPH SHOWS.

A further meeting of the Council of this Fund was held at the Mansion House last Tuesday, the Lord Mayor (Sir T. Vezey Strong) in the Chair.

The purpose of the meeting was to submit the further recommendation of the General Purposes Committee on the subject of Sunday cinematograph shows in support of hospitals.

The Archdeacon of London, as Chairman of the General Purposes Committee, moved the following resolution :—

"That the Council has fully considered the recommendations of the General Purposes Committee on the subject of Sunday cinematograph entertainments run by hospitals, as well as to the proposal of certain places of worship to ear-mark their collections for hospitals which do not participate in the proceeds of such shows, and while not expressing any opinion upon the expediency or otherwise of Sunday entertainments, resolves that in the event of the collections for the Fund suffering any diminution which may be fairly attributable to this action of certain hospitals, it shall be a recommendation to the Committee of Distribution to take the same into consideration when making their awards for the year.

"It is ordered that a copy of this Resolution be forwarded to the Committee of Management of each of the hospitals concerned."

### The Archdeacon's Speech.

In supporting the resolution, the Archdeacon reminded the Council that, through the courtesy of the meeting to Deputy Wallace, the matter was taken back by the Committee, the Council having thought that Mr. Sydney Holland had not been properly heard on the subject. Mr. Holland had now once more explained his views, and the Committee had modified their recommendations to the Council in the form now submitted. It was hoped that in this shorter form the views of the Council would be better understood. The Committee passed it unanimously with one exception, and it was urging nothing against the licensing of cinematograph shows on Sunday; they were not called upon to express any opinion. They had no wish to interfere with the free action of any hospital in the management of its own affairs. But the Committee claimed a like freedom in regard to the Hospital Sunday Fund in the management of its own delicate business. The bringing in of a bone of contention might endanger the very existence of that beneficent movement, in loyalty to which all were alike agreed. The proposal of the Committee was in accord with one of the Laws of the Constitution, Law IX., which provided that should any collection be made in a place of worship of which the Hospital Sunday Fund had not received the proceeds, that amount should be deducted from the grant made to such hospital. Nothing so drastic was now proposed; but if by the action of any hospital or hospitals prejudice had been raised against the Fund, and its income diminished thereby, that loss should be taken into consideration by the Distribution Committee. A number of charities had entered into an alliance with the owners of such shows to run their shows on Sundays for the purposes of charity, and to hand over their expenses. The allowance for expenses was sufficient to make it worth the owners' while to let out their premises in that way. The law was against the opening of any place of public amusement for profit on Sunday, and in the interests of theatres, music-halls, circuses, and the like, it was easy to see it was well that such permission should be withheld. But the London County Council said the shows might be run on Sunday if for a charitable purpose, the owners of such shows being paid therefor. Many charities had accepted that bargain. Amongst them were five hospitals : London, Poplar, Evelina, the Epileptic, and the Kingsland. Suggestions had been made to other hospitals, but they had declined to make use of the method. Three churches which stood in the front rank in London wrote to the Secretary of the Fund, saying they would not support the hospitals which used money so collected, and if nothing could be done to prevent it, they would ear-mark their contributions to the Hospital Sunday Fund. The Fund was an exceptional charity, in that it depended for its support and maintenance on the contributions of those who attend the various churches and synagogues of London, and the contributors must accordingly be credited with strong and definite religious convictions, and their ideas were entitled to attention. The churches were not important merely because they contributed large sums of money, but because of the character of their clergy and of their congregations. The Committee felt sympathy with the work carried on by Mr. Sydney Holland. The income of the London Hospital was £149,000, of which more than £100,000 was raised by Mr. Holland. But the Committee thought that if Mr. Holland were to discontinue that method of raising the £1,300 which was under discussion, he would not be the loser; those who were religious would be sure to prove their gratitude by giving increased sums.

### A Surgeon Supports the Resolution.

Sir Alfred Pearce Gould seconded the motion, and remarked that there had not been any proposal made by that Council or by the General Purposes Committee which bore any resemblance to the deprivation of hospitals running such shows from the benefits of the Fund. Four proposals were laid before the Committee. Mr. Sydney Holland's proposal was that the Council should take no action in the matter; but that the Committee thought would be a dereliction of duty. The second proposal was to ask the hospitals associated with the shows to make a return of the exact receipts they had every year, and that the grant they received from the Fund should be decreased by that precise sum. He was glad to say that that proposal found no support. The third proposal, which was of more importance, was that the Council should decline to express an opinion on the action of the hospitals in question, or to interfere in any way with the free action of the representatives ot any congregation making a collection on Hospital Sunday. The Committee regarded that as a very unfortunate suggestion, because it seemed to contain the germ of destruction of that great Fund. Churches forwent their liberty to hand their collections to one in which they felt special interest. The Fund was combined for a great purpose, and that great purpose involved the sacrifice of some individual liberty. The fourth proposal was the one now submitted. This only said that should it be made clear in the future that by this action of certain hospitals the Fund suffered, that fact should be considered by the Distribution Committee. Surely nothing could be fairer than that. The Council had nothing to do with the observance of Sunday; it had to secure the largest contributions for the hospitals, and to distribute it as fairly as it could.

Sir Henry Burdett said the sentiments of the speeches of the proposer and seconder of the resolution were not consistent with the wording of the resolution itself. For the latter clearly laid it down—if the resolution passed—that the Council disapproved of the action of the hospitals concerned in the free exercise of their own responsibilities. Therefore he strongly objected to the wording of the resolution. The responsibility of every member of the Council on the present occasion was greater than ever before, because on the wisdom or otherwise of its present action would largely depend the future prosperity of the Fund. The Archdeacon, not apprehending the exact circumstances, was unintentionally unjust to those hospitals which had heretofore exploited cinematograph shows. His point was that the Fund was a neutral body of trustees representing the congregations and the hospitals, and as such neutral Fund, it must be maintained as a centre of unity, and saved from becoming the centre of discordant voices. The resolution now brought forward by the General Purposes Committee clearly took sides; there was something in it of the nature of a threat that hospitals which took occasion to benefit by such Sunday shows were liable to be fined. That he did not regard as right or proper, nor safe as a matter of policy. Freed from all controversy, what remained was a matter of business. That business was that there were 97 hospitals, apart from cottage hospitals, receiving grants from the fund, and of those, five only were receiving money from cinematograph shows. Some might say it showed comparative lack of energy on the part of the managers; but might it not equally mean their wisdom? He knew there were some who had deliberately declined to employ that source of income. Sir Henry reviewed the conditions under which such shows at present could be held on Sundays. The average collection of the Hospital Sunday Fund was about £40,000 a year; but the total revenue for any Hospital from the Sunday cinematograph shows, under the limited conditions, must be less than £2,000 a year. The Hospital Sunday Fund had only five hospitals on the list which received a grant of over £2,000 a year from the Fund, so there were only five which, as a matter of business, could find it worth their while to open such shows on Sunday. It was not therefore good business for the hospitals, and in the future it would not be good business for the managers of these shows. He urged that, as a Council, they should "wait and see." Meanwhile the Fund should be kept outside this controversy, and hospitals and congregations should be left free to take their own course. He did not think there was the danger of reprisals which Mr. Gamble and others expressed their fear of at the last meeting, and Law IX. was an additional safeguard. He moved the following amendment: "That, though the Council fully recognises the right, first of hospital managers to run Sunday cinematograph entertainments as a source of income, and secondly, of congregations to earmark their collections to the exclusion of charities deriving income from Sunday cinematograph entertainments, they, as trustees to the hospitals and the congregations, for the present at any rate, declined to take any further action in the matter."

The Rev. Mr. Grundy seconded the amendment.

After considerable discussion Sir Henry Burdett, in order to simplify the issue, moved "The previous question." This was seconded by the Rev. Canon Curtis and lost.

The Hon. Sydney Holland, speaking to the original resolution, said that as the Council had been appealed to to take action in the matter by subscribers to the Fund, it was bound to send a courteous reply to them, one way or the other. He maintained that they were taking a very dangerous step in interfering with the matter; they were placing themselves in a controversy which disturbed the people on both sides. He again asked where such interference was to stop. His hearers would not get back the Cromwellian Sunday, however much they tried, and if the shows could not be run on Sunday for the benefit of hospitals, they would be run for less worthy objects. Clergy made a large assumption when they said that church-goers were all on their side; because they were not. If the Fund objected to the Sunday shows, let them go to the County Council and object to them; but do not let them try to stop money from going to the hospitals. One appealed for money for hospitals from both religious and irreligious people, and there were many religious people who saw no harm in those shows.

Lord Cheylesmore denied that the resolution interfered with any particular hospital; it simply protected those hospitals in case the funds this year should be diminished.

The Rev. Dr. Horton agreed that the great object to be aimed at was unanimity, and anything possible should be done to prevent serious division. If the object of the resolution was to allay the scrupulous consciences of certain congregations, he asked whether the present resolution was likely to have that effect. His fear was that the resolution, which was designed for a particular object, must accomplish something, but must fail in the object for which it was designed. The objection on the part of the churches was not that exception was taken to cinematograph shows, but because there was widespread anxiety in the country to preserve the English Sunday. That appeal was being made widely from many sources, and at the same moment this episode intervened, and the churches and the country wished to be reassured. He did not think the Council should hesitate to express its opinion on that point. The leading article in The Hospital refused to do so; it would not have any expression of opinion as to whether cinematograph shows should or should not be held on Sundays. This Council represented the churches and the synagogues of London, and consequently the religious forces of the Metropolis, and so it was not an extraneous body. If it were likely to lead to unanimity on the Council, he would propose the following amendment: "That this Council, while it is of opinion that the London County Council would do well, in view of the widespread anxiety to maintain the rest and quiet of our English Sunday, to refuse permission to Sunday cinematograph shows, does not consider it to be within its competency to penalise hospitals which avail themselves of this mode of raising income while the County Council licenses the shows for charitable purposes." If the amendment did not find favour, he would drop it.

The Hon. Sydney Holland seconded.

The Rev. Canon Curtis (Balham) supported it.

The Rev. Mr. Gamble opposed it, because it raised the question of Sunday observance which it had been several times promised should not be raised. He was not prepared to take up the position that all kinds of Sunday amusements were to be deprecated; it depended on the character of the amusements and the times at which they were held. These shows seemed to be of a harmless character; he visited one last Sunday, and it was dull but innocent.

The Rev. John Bradford thought that if Dr. Horton had been present at the recent meeting of the Council he would have hesitated to bring forward his present amendment. The Hospital had indicated the course which should be taken, but the Council must protect the Fund as far as possible, and that was what appealed to him more than anything. He thought the Fund would suffer by some hospitals running the shows which were under debate. It was to preserve the integrity of such a splendid Fund that he would vote for the resolution submitted by the General Purposes Committee.

The Rev. Dr. Munro Gibson suggested that as the unanimity which Dr. Horton hoped for was not apparent, he would withdraw his amendment.

Dr. Horton, however, as he had found a seconder, preferred to go to the vote. It was put to the vote, and lost, only six voting in its support.

Mr. Scriven submitted an amendment as follows:—"That the Council, after carefully considering the question of hospitals which receive money from Sunday cinematograph entertainments, finds itself unable to deal with the matter until it had been laid before a general meeting of the constituents of the Fund." He suggested that some compensation should be granted to those hospitals which had to discontinue that source of revenue.

No seconder being forthcoming, this fell to the ground.

The original resolution was then put, and carried, seven voting against.

### Other Business.

Mr. Norman reported that the small Sub-Committee had gone through the letters concerning the Battersea General Hospital, and read out the suggested reply to that hospital, dealing with all the points which had been raised. This was agreed to. The Secretary announced that a copy of a resolution had been received from the Rev. Mr. Shakespeare, Baptist Church House, Kingsway, which was passed by the Council of that body. The resolution expressed the opinion of the Council of the Baptist Union that the Hospital Sunday Fund would seriously suffer if the hospitals were the recipients of the proceeds of Sunday exhibitions.

This was ordered to be placed on the records, and the meeting then terminated.

